# Effects of online palliative care training on knowledge, attitude and satisfaction of primary care physicians

**DOI:** 10.1186/1471-2296-12-37

**Published:** 2011-05-23

**Authors:** Marta Pelayo, Diego Cebrián, Almudena Areosa, Yolanda Agra, Juan Vicente Izquierdo, Félix Buendía

**Affiliations:** 1 Primary care, NHS, Valencia, Spain; 2 Getafe University Hospital, NHS, Madrid, Spain; 3 Guadarrama's Hospital, NHS, Madrid, Spain; 4 Quality Agency of the NHS. Ministry of Health and Social Policy, Madrid, Spain; 5 Ribera's Hospital, Valencia, Spain; 6 Universidad Politécnica de Valencia, Spain

**Keywords:** palliative care, competency-based education, education continuing, medical informatics

## Abstract

**Background:**

The Spanish Palliative Care Strategy recommends an intermediate level of training for primary care physicians in order to provide them with knowledge and skills. Most of the training involves face-to-face courses but increasing pressures on physicians have resulted in fewer opportunities for provision of and attendance to this type of training. The effectiveness of on-line continuing medical education in terms of its impact on clinical practice has been scarcely studied. Its effect in relation to palliative care for primary care physicians is currently unknown, in terms of improvement in patient's quality of life and main caregiver's satisfaction. There is uncertainty too in terms of any potential benefits of asynchronous communication and interaction among on-line education participants, as well as of the effect of the learning process.

The authors have developed an on-line educational model for palliative care which has been applied to primary care physicians in order to measure its effectiveness regarding knowledge, attitude towards palliative care, and physician's satisfaction in comparison with a control group.

The effectiveness evaluation at 18 months and the impact on the quality of life of patients managed by the physicians, and the main caregiver's satisfaction will be addressed in a different paper.

**Methods:**

Randomized controlled educational trial to compared, on a first stage, the knowledge and attitude of primary care physicians regarding palliative care for advanced cancer patients, as well as satisfaction in those who followed an on-line palliative care training program with tutorship, using a Moodle Platform vs. traditional education.

**Results:**

169 physicians were included, 85 in the intervention group and 84 in the control group, of which five were excluded. Finally 82 participants per group were analyzed. There were significant differences in favor of the intervention group, in terms of knowledge (mean 4.6; CI 95%: 2.8 to 6.5 (p = 0.0001), scale range 0-33), confidence in symptom management (p = 0.02) and confidence in terms of communication (p = 0.038). Useful aspects were pointed out, as well as others to be improved in future applications. The satisfaction of the intervention group was high.

**Conclusions:**

The results of this study show that there was a significant increase of knowledge of 14%-20% and a significant increase in the perception of confidence in symptom management and communication in the intervention group in comparison with the control group that received traditional methods of education in palliative care or no educational activity at all. The overall satisfaction with the intervention was good-very good for most participants.

This on-line educational model seems a useful tool for palliative care training in primary care physicians who have a high opinion about the integration of palliative care within primary care. The results of this study support the suggestion that learning effectiveness should be currently investigated comparing different Internet interventions, instead of Internet vs. no intervention.

**Trial Registration:**

German Clinical Trials Register DRKS00000694

## Background

Palliative care (PC) in Spain can be delivered to patients at hospital and in the last months of life mainly at home, by primary care physicians (PCPs) supported by specialist PC teams, not equally distributed all over the country, and with access to an inpatient facility when required. The Spanish PC Strategy recommends an intermediate level of training for PCPs in order to provide them with knowledge and skills. Most of the training consists of face-to-face courses, but increasing pressures on PCPs have resulted in fewer opportunities for provision and attendance to this type of training.

Training needs of PCPs in PC have been described at great length in scientific literature [1-4]. A systematic review of educational methods in PC for PCPs showed that those educational activities with multi-faceted methodology offered better results than those using simple didactic approaches [5]. The only study using Internet as supporting element, together with a joint approach, showed significant outcomes in terms of palliative treatment choice, and regarding PCPs' attitude and satisfaction in the intervention group.

E-learning or learning facilitated and supported through the use of information and communication technologies offers a learner centred model consistent with the adult learning theory [6,7], where a direct and active learner involvement is required in order to obtain a subsequent behavior change. E-learning puts learners in control of their own learning in comparison with traditional instructor centred teaching. On-line educational programs offer practical benefits for learners who work well with computers and have access to Internet. It provides a real alternative to traditional methods of education because of its flexibility and interaction capabilities and is rapidly gaining in importance [8]. The efficacy of on-line continuing medical education is already quite well established in two aspects: knowledge acquisition, with results comparable to other traditional models, and learner satisfaction, with higher satisfaction results compared to other models. However, there is uncertainty in terms of potential benefits of asynchronous communication and interaction among participants, and the effect of the learning process. Asynchronous communication, or the relay of information with a time lag, e.g. in discussion forums and e-mails, provides benefits for learning. Some proposed benefits are: that it helps learners to internalize and process information, it offers all learners equal opportunities to participate, it needs a lower hardware and network specification and it is very flexible. The main disadvantages to asynchronous communication are the waste of time while waiting for a response, and the learner's isolation [9].

There has been less research in terms of the effectiveness of on-line continuing medical education, in order to identify whether said model has an impact on clinical practice; and those few known results are mostly based on the physicians' own perception, instead of objective clinical measurements; therefore, these outcomes are still uncertain [10-13]. Data from a systematic review [14] and from other studies [15] have detected discreet improvements of 4%-5%, or none at all. In the topic of PC and cancer the on-line model has also been successfully used for end-of-life care consultation [16], for pain management [17], or to obtain clinical information [18]. However, the effectiveness of on-line PC training for PCPs is currently uncertain, regarding improvement in patient's quality of life and main caregiver's satisfaction, which points out to the need of a clinical impact assessment through objective measurements [5,19].

On-line continuing medical education is promising in terms of its ability to improve clinical practice, as long as the information provided, dissemination planning and pedagogical approach are accessible, useful, high quality, and focused on the learner [12,20]. To determine its effectiveness in PC training for PCPs is important due to the fact of increasing constrains of attendance to face to face training and the quickly development of new technologies applied to instruction.

Planning and design of on-line training is particularly complex, as it involves multidisciplinary teams working together in order to shape educational materials according to the chosen pedagogical approach. Didactic resources must be organized and scheduled, tutor-mediated interaction between learners and teaching staff must be planned, and satisfaction and efficacy in terms of learning must be provided.

The authors have developed an on-line educational model for PC which has been applied to PCPs to measure its effectiveness regarding knowledge, attitude towards PC, perception of confidence of symptom management and communication with advanced cancer patients requiring PC and their relatives, and PCPs' satisfaction, in comparison with a control group and random allocation.

The effectiveness evaluation at 18 months, through objective clinical measures, of the impact on the quality of life of patients managed by the physicians, and the main caregiver's satisfaction will be addressed in a different paper.

## Methods

Experience Setting: the training model being assessed has been applied during 2009 to PCPs from primary care centers in Spain. Participants were recruited by informing specific groups about the project, and offering teaching credits, participation as collaborator in the final publication of patients' results and financial incentives (80 euros per patient included). Participants had to be: PCPs from the Spanish NHS (with a practice of their own, substitute position ≥ two years, acceptable level of English in order to read medical texts, and Internet access). Those PCPs included were randomized (the physician is the unit of randomization) in two groups by computer-generated exchangeable sequence of two-blocks kept in opaque envelopes. Allocation was conducted when PCPs agreed to take part, by a researcher who didn't know the random sequence, and a sequential numerical code was then assigned. The intervention group had access to the on-line program for PC self-training, while the control group did not, but could voluntarily receive or not the usual PC training offered for his working area (traditional training).

**Figure 1:**
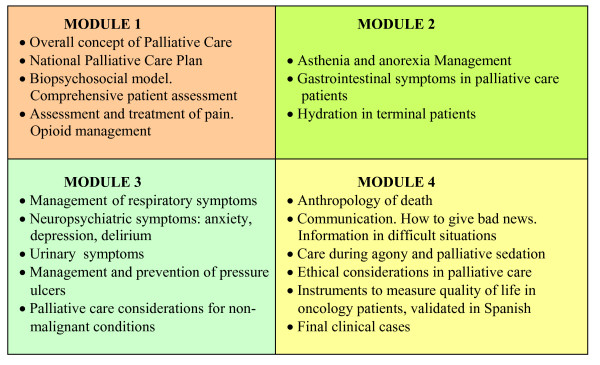
**Contents of Didactic Modules for Palliative Care**. Educational content of palliative care and the distribution of modules

Educational Contents: it was prepared by the authors according to clinical competences of PCPs in PC, as recently defined in the specialty program [21], and distributed in four modules (Figure 1). These modules were divided into subjects including: objectives, contents directed to clinical practice, with clear and concise explanations, PC bibliography and websites. These websites were chosen according to a previous pilot study, in which 27 PCPs assigned a score to the best websites based on their clinical usefulness, the quality of their information, and their accessibility. Each subject included links to explanations for the presentation, and self-guided questions which could be reviewed several times, without a final score.

Technological Platform: E-Learning platforms, also called VLE (Virtual Learning Environments) or LMS (Learning Management Systems), allow access to instructional information from any location and any time by using a personalized identification, making its use compatible with other activities. At the moment, there is no clear differential concept of technological platforms that facilitate learning and the variety of denominations are defined in terms of their functionality or a description of their functions and tools, instead of a definition consistent with the final objectives of the platform. All these platforms use a system or combination of rules, process or workflows that effectively manages any type of digital information, whether text, images, video, documents, sound files, etcetera. The main concept behind any Content Management System is to make the digital information available for inter-office or on-line edition.

**Figure 2:**
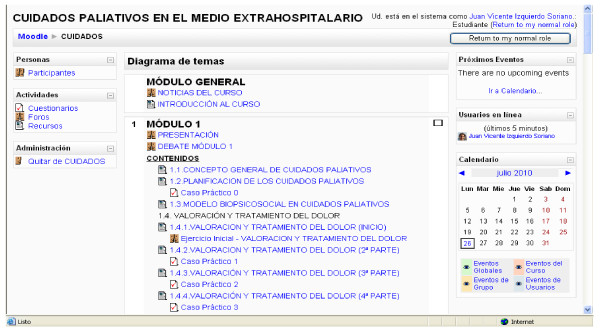
**Moodle Platform**. General aspect of the educational content in the Moodle platform

Moodle is a very popular e-learning platform, which is widely used in health education contexts because it allows instructors to prepare and store educational resources easily, in order to deliver them to potential users. It was selected for our study in order to provide physicians with a common repository for the educational resources used in the current experience [22-24]. The Dreamweaver tool was used for content design, and the platform resources (Figure 2) were prepared based on Word documents. Evaluation forums and questionnaires were conducted, complemented by other interactive resources (images, diagrams, videos, interactive webpages, among others).

Assessment: The quality of on-line training process, knowledge, and attitude towards PC were assessed, as well as satisfaction with the teaching activity.

Variables registered on-line were:

### -Professional data

Age, gender, number of years working in Primary Care, specialty, Medical Residency Training, number of patients requiring PC/year on average (0 to 10, numerical and >10), PC training during the last five years.

### -Attitude towards PC

Questionnaire prepared by the authors and previously used in the aforementioned pilot study, with 5-point Likert-scale answers. The perception of support by the area PC Team was explored, as well as the perception of confidence in symptom management, perception of confidence in communication, and a score was assigned to the statement: "PC for advanced cancer patients should be part of care offered by PCPs."

### -Basic knowledge

A 33-item questionnaire with a single correct answer. The score consisted of the arithmetic sum of all correct items (with a maximum score of 33). This questionnaire was prepared by the authors following the recommendations about post-graduate medical education assessment [25], and tested in 12 PCPs from different primary care centers, to ensure the validity of its contents and those required modifications to facilitate its understanding.

The intervention group had two tutors available to answer to any doubt through the platform and forums, thus facilitating communication among learners. On-line training, lasting 96 hours, and with 15 credits, was conducted during 75 days (March to May, 2009), with 15-20 days per module, though the platform could be accessed during the whole study.

Evaluation was conducted through a second knowledge questionnaire, similar to the baseline one, as well as the same attitude questionnaire (confidence in symptom management and communication). The intervention group was asked a satisfaction questionnaire, based on the above mentioned pilot study, and another Moodle evaluation questionnaire, with more technical platform aspects (Tables 1, 2). PC training outside the study was registered, as well as group contamination.

**Table 1:** Process Aspects Assessed in On-line Education

ASPECTS ASSESSED	LIKERT SCALE
1. The QUALITY of information within the educational materials is	
	
2. The TIME devoted to each module has been	
	
3. The PRACTICAL USEFULNESS of the educational contents is	Very poor, poor, acceptable, good, very good
	
4. The SYSTEMATIC process for on-line education has been	
	
5. Overall, the DIFFICULTY of on-line education has been	
	
6. TUTOR SUPPORT has been	

7. QUESTIONS asked in forums have been answered satisfactorily by tutors	Strongly disagree, disagree, acceptable, agree, strongly agree

8. OVERALL SATISFACTION is	Very low, low, acceptable, high, very high

9. I WOULD RECOMMEND my colleges to take part in this activity	Strongly disagree, disagree,
	
10. If there was a future on-line training about any specific clinical aspect using the same systematic approach as this activity, I WOULD TAKE PART	acceptable, agree, strongly agree

11. How would you describe YOUR CONFIDENCE in symptom management for advanced cancer patients requiring palliative or terminal care?	Very poor, poor, acceptable,
	
12. How would you describe YOUR CONFIDENCE in communication with advanced cancer patients about disease diagnosis and prognosis?	good, very good

13. Please briefly describe the most useful aspects	
	
14. Please briefly describe any aspects you would add	Open
	
15. Please briefly describe any aspects you would remove	
	
16. Other comments	

Satisfaction and self-confidence assessed to the participants of the on-line training

**Table 2:** Technical Aspects Assessed in the Moodle Survey

1 - Do you consider the use of the Moodle Platform adequate to achieve said objective?
2 - Do your skills match those required to use the Moodle Platform?
3 - Do you think the amount of time and effort required to use Moodle is compensated by those results achieved in terms of reaching the objectives?
4 - Do you think that integrating the Moodle Platform in the course leads to an improvement in the activity?
5 - Has it been easy for you to use or access those resources available in the Moodle Platform?
6 - Do you think those resources available in the Moodle Platform are enough to carry out the intended activity?
7 - Do you think that using the Moodle Platform facilitates conducting the activity in an organized manner?
8 - Do you consider that instructions and other pieces of information provided through the Moodle Platform have been clear and accurate?
9 - Do you think that the use of the Moodle Platform has allowed you to answer with higher security the activity assessment questions?
10 - Do you consider that using the Moodle Platform has improved your acquisition of theoretical knowledge in terms of the future overall evaluation?

Aspects evaluated in the Moodle survey

(Answers in Likert Scale: Completely Disagree, Disagree, Neutral, Agree, Completely Agree)

A minimum of 60 PCPs per group was calculated, for a score difference of 20% in the short-form pain questionnaire (used to measure impact upon patients) among groups, 5% significance, 90% power, and 10% loss calculations. Frequencies and mean comparisons were analyzed with SPSS (version 18), non-parametric tests were conducted (sign test and Mann-Whitney U test for matched and independent ordinal data) with significant bilateral contrasts in level 0.05, and the Cohen's effect size was calculated between both groups. Group allocation was maintained using multiple imputation for missing values.

## Results

The study involved 169 PCPs throughout the whole country, with 85 in the intervention group and 84 in the control group. Five participants (three from the intervention group, two from the control group) were excluded for not providing data; they were three men and two women, all of them on payroll except one substitute. Finally 82 participants per group were analyzed. Baseline characteristics (Tables 3, 4) were balanced with a minimal difference in terms of previous PC training in the intervention group (78%) vs. the control group (70%). Most participants were family doctors (through Medical Residency Training), women, age > 40 years, wide professional experience, on payroll, with recent PC training, and most of them seeing two-four patients per year requiring PC. Most participants had support by the area PC Team, and its performance was considered from good to very good; their perception of confidence in symptom management was acceptable-good, as well as confidence in communication; they agreed-highly agreed that PC for advanced cancer patients should be part of care offered by PCPs.

**Table 3:** Study Participant Characteristics

CHARACTERISTIC	INTERVENTION (N = 82)	CONTROL (N = 82)
	M (SD)	M (SD)

**Age**	48 (6)	47 (6)

**Years working in primary care**	20 (6.4)	18 (6.6)

**Test of previous knowledge **(maximum score: 33) ¶	20.3 (3.3)	19.9 (2.6)

**Gender**	n (%)	n (%)
Male	38 (46)	36 (44)
Female	44 (54)	46 (56)

**Employment status **Full time	70 (85)	70 (86)
Temporary	12 (15)	12 (14)

**Medical Residency Training**	58 (71)	55 (65)

**Specialty**		
- Family Medicine	77 (94)§	75 (91)*
-Others	0	1 (1)
-None	5 (6)	6 (7)

**Completion of any educational activity regarding palliative care during the last 5 years**	64 (78)	59 (70)

**No. of palliative care patients seen per year**		
0-1	4 (5)	2 (2)
2-4	55 (67)	52 (63)
5-10	22 (27)	22 (27)
>10	1 (1)	6 (7)

Baseline characteristics of the participants included in the study

¶ refers to 79 participants in the intervention group and 72 in the control group; § 3 participants had another specialty besides Family Medicine (Gastroenterology 1, Haematology 1, Nephrology 1); * 5 participants had another specialty besides Family Medicine (1 Psychology, 2 Internal Medicine, 1 Nephrology and Public Health, 1 Psychiatry).

**Table 4:** Study Participant Baseline Characteristics (continued). Absolute Numbers and Value Percentages in Likert Scale.

Characteristic	Very poor		Poor		Acceptable		Good		Very good	
	Intervention/Control		Intervention/Control		Intervention/Control		Intervention/Control		Intervention/Control	
Support by the Palliative Team in the area *	7 (8)	3 (4)	1 (12)	6 (7)	15 (18)	9 (11)	22 (27)	25 (30)	27 (33)	27 (33)

**Level of Medical English (reading)**	7 (8)	4 (5)	17 (21)	16 (19)	29 (35)	37 (45)	21 (26)	23 (28)	8 (10)	2 (2)

**Confidence in symptom management for advanced cancer patients requiring palliative care**	0	0	12 (15)	14 (17)	41 (50)	43 (52)	28 (34)	23 (28)	1 (1)	2 (2)

**Confidence in communication with advanced cancer patients about disease diagnosis and prognosis**	1 (1)	1 (1)	12 (15)	10 (12)	36 (44)	36 (44)	32 (39)	29 (35)	1 (1)	6 (7)

	**Highly disagree**		**Disagree**		**Neutral**		**Agree**		**Highly agree**	

**Opinion about "Palliative care for advanced cancer patients should be part of care offered by the primary care team."**	0	1 (1)	3 (4)	2 (2)	0	1 (1)	43 (52)	26 (32)	36 (44)	52 (63)

Continuation of baseline characteristics of the study population

10 (12%) in the intervention group and 12 (15%) in the control group stated there is no Palliative Care Team in their area.

### Evaluation of Access to the On-line Platform

Out of 82 intervention group participants, 59 (72%) completed all modules, 12 (14.6%) didn't complete them, and 11 (13.4%) never accessed the platform. This sub-group of 23 (eight of which abandoned the study due to personal reasons), differentiated itself from those completing the modules by: being younger, less on payroll, and fewer family doctors. The 11 who never accessed the platform were mostly male with less Medical Residency and PC training. Nine were supported by the area PC team and perceived their self-confidence in symptom management between acceptable and good; all of them scored their self-confidence in communication between acceptable and good, and except one all agreed-highly agreed that PC for advanced cancer patients should be part of care offered by PCPs. Their mean score in knowledge was 19.2 (SD 2.8).

The average number of visits to the platform was 266.8 visits (SD 87.3; range 118-509) for those completing modules, and 56.2 (SD 58; range 8-189) for those who did no. Forty-two participants (59%) took part in the discussion forum.

Within the control group, 11 (13.4%) participants received PC training: hospital rotation in palliative unit (1), personal attendance courses (3), on-line course (1), self-study (5) and clinical session (1). These were all short-duration (around 20 hours), and only included basic PC aspects with less specific contents in terms of pain control, communication and quality of life.

### Moodle Platform Evaluation

**Figure 3:**
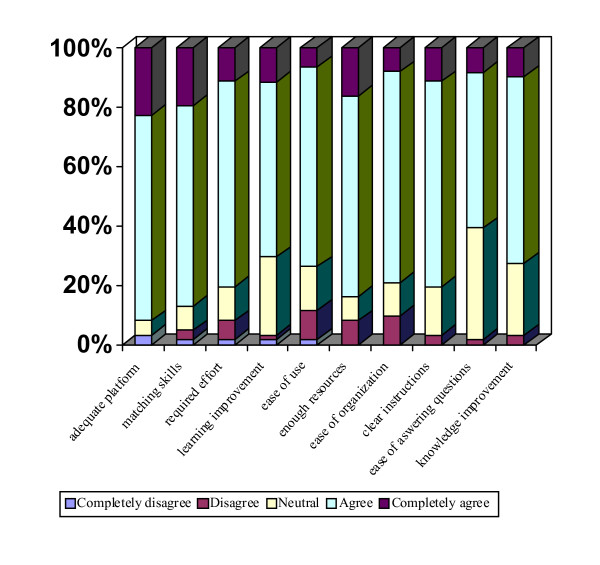
**Overall Score for the Moodle Platform Survey**. Results of the technical evaluation of the platform

Sixty two (75.6%) survey forms about the platform were received. The most frequent score (Figure 3) was "agree", from 52.5% in question 9 (*Do you think that the use of the Moodle Platform has allowed you to answer with higher security the activity assessment questions?*), to 71% in question 7 (*Do you think that using the Moodle Platform facilitates conducting the activity in an organized manner?*). Twenty-seven participants (43.5%) made comments regarding: on-line application difficulties (communication in the forum, difficulties with the platform, sending of answers of questionnaires), or subject presentation (few graphs, subjects too segmented, lack of materials in pdf format).

### Evaluation of Knowledge, Attitude and Satisfaction

**Figure 4:**
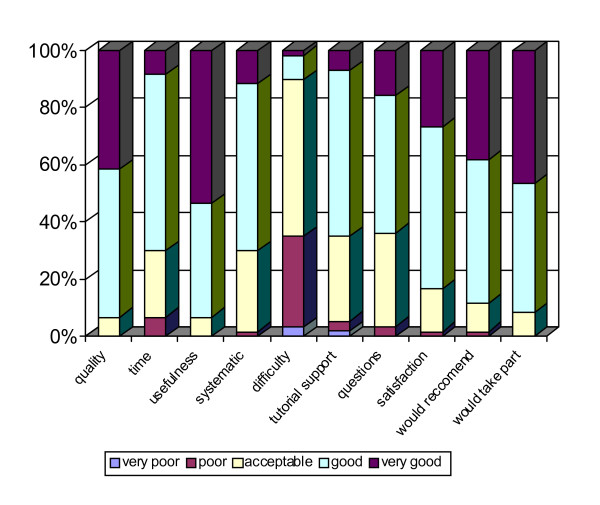
**Overall Score for the Satisfaction Questionnaire**. Results of the evaluation of satisfaction with the on-line training

One hundred thirty one (80%) questionnaires were received, corresponding to 60 (73.2%) intervention group participants, including activity satisfaction; and to 71 (86.6%) control group participants. The most frequent score (Figure 4) was "good" or "very good" with a score of "poor" or "acceptable" the difficulty of conducting on-line training.

In order to maintain intent to treat in the analysis missing values were examined and concentrated in five variables, in 35 (21.3%) cases (22 intervention and 13 control). Said examination indicated that they were not missing completely at random; therefore, five multiple imputations were conducted. The results in knowledge, confidence in symptom management and confidence in communication are shown in the Table 5.

**Table 5:** Results in knowledge and confidence in symptom management and communication

Aspect assessed	Intervention	Control	Group Differences *(IC 95%) p*
Previous knowledge *mean (SD)*	20.3 (3.3)	19.9 (2.6)	0.4 (-0.5 to 1.4) NS

Posterior knowledge *mean (SD)*	25.4 (3.7)	18.1 (3.2)	7.3 (6.2 to 8.5)* *original* 5.2 (3.4 to 6.9)* *imputated*

*Original Data*			
Posterior - previous knowledge *mean (IC 95%)*	4.8 (3.6 to 5.9)*	-1.7 (-1.0 to -2.5)*	6.5 (5.2 to 7.9)* Effect size 1.7 (20%)
*Imputated Data*			
	3.0 (1.5 to 4.6)*	-1.6 (-0.3 to -3.0)	4.6 (2.8 to 6.5)*
	p = 0.035		Effect size 0.70 (14%)

Confidence in symptom management posterior: improvement of categories	1-3: 33.3% p = 0.151	1-2: 18.3%*	Effect size: 0.7 *original* 0.4 *imputated *p = 0.02

Confidence in communication: improvement of categories	1-3: 35% p = 0.007	1: 7% NS	Effect size: 0.5 original 0.3 imputated p = 0.038

Results in knowledge and confidence in symptom management and communication by group and differences among groups

*p = 0.0001 NS = no significative

Significant differences among groups in favor of the intervention group were found in knowledge, confidence in symptom management and confidence in communication.

The intervention group showed a significant increase in knowledge and confidence in communication, but not in confidence in symptom management. Eight (13.3%) participants of this group received PC training outside this study: two of them, within the hospital palliative unit, one took a presence course, and the rest received other types of training. Within this sub-group, the median post-test knowledge was 24 (SD 4.6) in comparison with their group that was 25.4 (SD 3.7) that is 4.2% of difference.

The control group decreased knowledge and increase confidence in symptom management in a significant way while the change in confidence in communication was non significant. The subgroup of 11 (13.4%) that reported some kind of education in PC had a post-test knowledge score of 16.9 (SD 3.1) in comparison with 18.1 (SD 3.2) in their group, that is 3.6% of difference.

The *most useful aspects *pointed out in terms of on-line training (50 participants (83.3%)) were: practical, clear and systematic approach, with elaborated and updated materials; symptom management, death management, communication, opioid management; bibliography and Websites; tutoring and communication among participants. The *aspects to be improved *in terms of training, pointed out by 42 participants, were associated with computer program design, platform management, or specific subject aspects.

There was no group contamination.

The resources needed for the preparation and implementation of this training were four months for the elaboration of educational contents, one hour a week for three months for tutoring the participants, and about 10 hours for administrative tasks in all procedures. Participants spent about 96 hours in training. A total of 6.500 € were allocated for the on-line adaptation of the educational material. Teachers were the project researchers, so they did not receive any specific budget.

## Discussion

The outcomes of the first stage of this study have shown that, after an on-line PC training program targeted to PCPs, using a Moodle Platform, with tutoring, and developed during 75 days, there was an increase of knowledge of 14%-20%, there was a significant increase in the perception of confidence in symptom management and communication, and overall satisfaction was good-very good for most participants. The cost for the education consisted of time spent by the authors in the implementation of the program and logistics. We value the time spent as very positive if we consider that the education model can be offered to other physicians interested in PC education in the near future.

These outcomes are comparable to other studies for on-line educational intervention, such as those reported about pain management and other oncology symptoms, or management of last hours of life [8-12,26-29]. Most participants assigned a score of "good-very good" to the quality and usefulness of educational contents, with recommendation to other colleagues to take part, and agreement that the integration of platform resources facilitated the learning process. Even though some technical aspects to be improved were pointed out, the overall difficulty in terms of using the platform was considered low.

The decrease in knowledge in the control group, and specifically in the sub-group of 11 who received some kind of education, could be explained by the fact that the majority did not report any educational activity, and only four participants of 11 attended courses with more extensive subjects, while the rest reported self-study, perhaps with more emphasis on specific subjects, not so wide as those explored by the knowledge test. Regression to the mean could be another explanation. The probability of chance is low.

Confidence in communication increased significantly in one to three categories in the intervention group, with a marginal significance in the comparison among groups and low effect size in the data imputated. The subject of communication with the patient and the caregiver was elaborated through visual sketches and specific sentences that, in spite of being short in extension, could have made an impact on the confidence of the participants.

These results must be interpreted within the context of PCPs participating in this study with wide professional experience, knowledge, confidence in terms of symptom management and patient communication in PC from acceptable to high, frequently supported by the local PC team, and with a very high opinion about the integration of PC within Primary Care. Results could not necessarily be reproduced in another PCP profile, particularly in those with a low opinion about the integration of PC within primary care, and who are probably not able to read medical texts in English.

The variety of opinions by PCPs in terms of the on-line design of that training provided might be associated with different learning styles. Some studies about on-line training for healthcare professionals [30] suggest that those with an analytic learning style tend to respond better to less structured, long Web-pages, with an in-depth instead of a wide approach; on the other hand, those professionals with a more holistic style may respond better to less structured educational materials, shorter web-pages, and with a wider instead of an in-depth approach. It has been suggested that on-line education could be significantly encouraged by adapting teaching methods to analytic-holistic learning styles; this has not been thoroughly investigated in terms of medical education.

Another aspect about which there is limited knowledge is the emotional impact of terminal patient care, and how PCPs manage the relationship with patients and their family. Although this aspect was included in module 4 in our study, no specific questions came about from any participants about this issue. The study of this specific topic through a qualitative methodology, as has been carried out in other related topics [31], would allow us to include a future on-line module regarding emotional needs of PCPs, and tackle the issue at the same time.

Education using e-learning platforms has a highly positive and consistent effect when compared with no educational intervention, and seems to have the same efficacy and effectiveness than the traditional training system. A meta-analysis comparing medical knowledge acquired through internet vs. no intervention reported a considerable benefit in favor of Internet [32]. Even though these outcomes might be affected due to confusing factors or chance, it has been suggested that learning effectiveness should be currently investigated comparing different Internet interventions, instead of Internet vs. no intervention.

On-line PC training is increasingly spreading [33], and the on-going studies [34-36] will help to clarify its effectiveness. In our study, on-line training has been effective in improving PCPs' knowledge and attitude, and therefore we consider it a useful training tool.

### Study Limitations

The effect upon these results caused by external PC training received by eight participants from the intervention group, that supposes 4.2% of the total score, could affect the evaluation of differences among groups. Nevertheless these situations could be present in studies evaluating effectiveness.

Missing data from 22 intervention participants and 13 control participants are within those reported by scientific literature [32]. This is the strongest limitation, and it somehow suggests that those incentives offered might not have been attractive enough or the physicians did not feel the necessity to follow this specific program.

We have tried to make up for this deficiency by multiple imputation, establishing a comparison with original data and showing more conservative data.

## Conclusions

PC training for PCPs using an on-line educational model with a Moodle Platform and tutorship, showed an increase of knowledge of 14%-20%, and a significant increase in the perception of confidence in symptom management and communication, in comparison with a control group that received traditional methods of education or no educational activity at all. PCPs satisfaction was high.

According with the results this on-line educational model seems a useful tool for PC training in PCPs who have a high opinion that PC should be integrated in primary care, able to read English medical texts and supported at large by the PC team. Some limitations signaled are related with the current situation of medical practice.

The results of this study support the suggestion that learning effectiveness should be currently investigated comparing different Internet interventions, instead of Internet vs. no intervention.

The second phase of this study will examine on-line training effectiveness at 18 months, and its impact upon patient's quality of life and caregiver's satisfaction.

## Competing interests

The authors declare that they have no competing interests.

## Authors' contributions

P-AM: conceived of the study, and participated in its design, coordination and statistical analysis and helped to draft the manuscript. CD: participated in its design and elaboration of the educational material and tutorship. AA: participated in its design and elaboration of the educational material and tutorship. A-VY: participated in the study design and elaboration of the educational material. IJV: organized the online platform. BF: organized the online platform. All authors read and approved the final manuscript.

## Pre-publication history

The pre-publication history for this paper can be accessed here:

http://www.biomedcentral.com/1471-2296/12/37/prepub

## References

[B1] Salinas AM Asensio AF Armas J Benítez del Rosario MA Palliative care in primary care: professionals' opinion Aten Primaria 1999 23 187 91 10333601

[B2] Esteva MC Llobera JC Miralles JX Bauza MA Management of terminal cancer patients: attitudes and training needs of primary health care doctors and nurses Support Care Cancer 2000 8 464 71 10.1007/s005200000157 11094991

[B3] Barclay S Todd Ch Grande G Lipscombe J Controlling cancer pain in primary care: the prescribing habits and knowledge base of general practitioners J Pain Symptom Manage 2002 23 283 92 10.1016/s0885-3924(02)00389-5 12007756

[B4] Shipman C Addington-Hall J Barclay S Briggs J Cox I Daniels L Millar D Educational opportunities in palliative care: what do general practitioners want? Palliat Med 2001 15 191 6 10.1191/026921601678576176 11407190

[B5] Pelayo-Alvarez M Agra Y Systematic review of educational interventions in palliative care for primary care physicians Palliat Med 2006 20 673 83 10.1177/0269216306071794 17060266

[B6] Slotnick HB Physicians' learning strategies Chest 2000 118 18S 23S 10.1378/chest.118.2_suppl.18S 10939995

[B7] Grau-Perejoan O Formacion on line EDUC MED 2008 11 139 46

[B8] Curran VR Fleet L A review of evaluation outcomes of web-based continuing medical education Med Educ 2005 39 561 67 10.1111/j.1365-2929.2005.02173.x 15910431

[B9] Chumley-Jones HS Dobbie A Alford CL Web-based learning: sound educational method or hype? A review of the evaluation literature Acad Med 2002 77 10 Suppl 86 93 10.1097/00001888-200210001-00028 12377715

[B10] Wutoh R Boren SA Balas EA E-learning: a review of Internet-based continuing medical education J Contin Educ Health Prof 2004 24 20 30 10.1002/chp.1340240105 15069909

[B11] Casebeer L Kristofco RE Strasser S Reilly M Krishnamoorthy P Rabin A Zheng S Karp S Myers L Standardizing evaluation of on-line continuing medical education: physician knowledge, attitudes, and reflection on practice J Contin Educ Health Prof 2004 24 68 75 10.1002/chp.1340240203 15279131

[B12] Curran V Lockyer J Sargeant J Fleet L Evaluation of learning outcomes in Web-based continuing medical education Acad Med 2006 81 10 Suppl S30 S34 17001130 10.1097/01.ACM.0000236509.32699.f5

[B13] Weston CM Sciamanna C Nash D Evaluating online continuing medical education seminars: evidence for improving clinical practices Am J Med Qual 2008 23 475 83 10.1177/1062860608325266 19001103

[B14] Shojania KG Jennings A Mayhew A Ramsay C Eccles M Grimshaw J Effect of point-of-care computer reminders on physician behaviour: a systematic review CMAJ 2010 182 5 E216 E225 20212028 10.1503/cmaj.090578 PMC2842864

[B15] Fordis M King JE Ballantyne CM Jones PH Schenider KH Spann SJ Greenberg SB Greisinger AJ Comparison of the instructional efficacy of Internet-based CME with live interactive CME workshops. A randomized controlled trial JAMA 2005 294 1043 51 10.1001/jama.294.9.1043 16145024

[B16] Klueber KK Bruera E Interactive collaborative consultation model in end-of-life care J Pain Symptom Manage 2000 20 202 9 10.1016/S0885-3924(00)00176-7 11018338

[B17] Thompson AR Savidge MA Fulper-Smith M Strode SW Testing a multimedia module in cancer pain management J Cancer Educ 1999 14 161 3 10512333 10.1080/08858199909528608

[B18] Pereira J Bruera E Quan H Pallaitive care on the net: an online survey of health care professionals J Palliat Care 2001 17 41 5 11324184

[B19] Cauffman JG Forsyth RA Clark VA Foster JP Martin KJ Lapsys FX Davis DA Randomized controlled trials of continuing medical education: what makes them most effective? J Contin Educ Health Prof 2002 22 214 21 10.1002/chp.1340220405 12613056

[B20] Peterson MW Galvin JR Dayton Ch D'Alessandro MP Delivering pulmonary continuing medical education over the Internet Chest 1999 115 1429 36 10.1378/chest.115.5.1429 10334164

[B21] Programa de la especialidad de Medicina Familiar y Comunitaria Comisión nacional de la especialidad 2002 Madrid: Ministerio de Sanidad y Consumo20626584

[B22] EHS E-learning Health Scotland: e-Hospital e-learning while in hospital 2008 http://www.ehospital-project.net/

[B23] HUSC Hospital Universitario San Cecilio Granada 2008 http://www.juntadeandalucia.es/servicioandaluzdesalud/hsc/moodle/

[B24] PHT Portsmouth Hospitals NHS Trust 2008 http://www.i-am-in-the-moodle.co.uk/

[B25] Brown G Atkins M Effective teaching in higher education 1991 Ed: London Routledge repr

[B26] Koczwara B Francis K Marine F Goldstein D Underhill C Olver I Reaching further with online education? The development of an effective online program in palliative oncology J Canc Educ 2010 10.1007/s13187-009-0037-6 20119693

[B27] Arenella Ch Yox S Eckstein DS Ousley A Expanding the reach of a cancer palliative care curriculum through web-based dissemination: a public-private collaboration J Canc Educ 2010 10.1007/s13187-010-0066-1 20237885

[B28] Casebeer L Engler S Bennett N Irvine M Sulkes D Deslauriers M Zhang S A controlled trial of the effectiveness of internet continuing medical education BMC Medicine 2008 6 37 10.1186/1741-7015-6-37 19055789 PMC2612689

[B29] Leong L Ninnis J Slatkin N Rhiner M Schroeder L Pritt B Kagan J Ball T Morgan R Evaluating the impact of pain management education on physician practice patterns-a continuing medical education outcomes study J Canc Educ 2010 10.1007/s13187-010-0040-y PMC3751402 20204577

[B30] Cook DA Learning and cognitive styles in Web-based learning: theory, evidence and application Acad Med 2005 80 266 78 10.1097/00001888-200503000-00012 15734809

[B31] van Marwijk H Haverkate L van Royen P Anne-Mei T Impact of euthanasia on primary care physicians in the Netherlands Palliat Med 2007 21 609 14 10.1177/0269216307082475 17942499

[B32] Cook DA Levinson AJ Garside S Dupras DM Erwin PJ Montori VM Internet-based learning in the health professions: a meta-analysis JAMA 2008 300 1181 96 10.1001/jama.300.10.1181 18780847

[B33] Kevin Mc Launch of a new online training program to enhance palliative training in rural areas Aust J Rural Health 2007 15 389 17970904 10.1111/j.1440-1584.2007.00936.x

[B34] Kuziemsky CE Weber-Jahnke JE Lau F Downing M An interdisciplinary computer-based information tool for palliative severe pain management J Am Med Inform Assoc 2008 15 374 82 10.1197/jamia.M2519 18308992 PMC2410007

[B35] Street AF Swift K Annells M Woodruff R Gliddon T Oakley A Ottman G Developing a web-based information resource for palliative care: an action-research inspired approach BMC Medical Informatics & Decision Making 2007 7 26 10.1186/1472-6947-7-26 17854509 PMC2194759

[B36] Murray MA O'Connor A Stacey D Wilson KG Efficacy of a training intervention on the quality of practitioner's decision support for patients deciding about place of care at the end of life: A randomized control trial: Study protocol BMC Palliative Care 2008 7 4 10.1186/1472-684X-7-4 18447916 PMC2396601

